# Phospholipase D1 Ameliorates Apoptosis in Chronic Renal Toxicity Caused by Low-Dose Cadmium Exposure

**DOI:** 10.1155/2020/7091053

**Published:** 2020-03-31

**Authors:** Ke Huang, Yaotang Deng, Wenya Yuan, Jian Geng, Guanghai Wang, Fei Zou

**Affiliations:** ^1^Department of Occupational Health and Occupational Medicine, School of Public Health, Southern Medical University, Guangzhou 510515, China; ^2^The First People's Hospital of Zhaoqing City, Zhaoqing 526000, China; ^3^Department of Pathology, Southern Medical University, Guangzhou 510515, China

## Abstract

Exposure to cadmium (Cd), a common heavy metal used in industry, can result in long-term chronic toxicity. It has been well characterized that kidneys are the main organs that are targeted by toxicity, which can cause apoptosis, necrosis, and atrophy of renal tubular epithelial cells. However, the molecular mechanisms associated with Cd toxicity remain unclear. In this study, the expression of renal proteins in Sprague-Dawley rats exposed to chronic Cd was analyzed with iTRAQ proteomics. Bioinformatics analysis indicated that phospholipase D1 (PLD1) was significantly underexpressed and may correlate strongly with Cd-induced chronic kidney impairment. Previous studies have shown that PLD1 promotes cell proliferation and inhibits apoptosis, indicating that PLD1 may be implicated in the pathogenesis of kidney injury induced by Cd. Studies *in vivo* and *in vitro* all demonstrate that the mRNA and protein levels of PLD1 decrease significantly both in kidney tissue and in proximal tubular cell lines exposed to Cd. Overexpression of PLD1 and its downstream product PA could ameliorate Cd-induced apoptosis. Moreover, we identified that miR-122-5p was a regulatory miRNA of PLD1. miR-122-5p was overexpressed after Cd exposure and promoted cell apoptosis by downregulating PLD1 through binding the 3′UTR of the locus at 1761–1784 nt. In conclusion, our results indicated that PLD1 and its downstream PA were strongly implicated in Cd-induced chronic kidney impairment and could be a novel player in the defense against Cd-induced nephrotoxicity.

## 1. Introduction

Cadmium, a common heavy metal in industry, has the potential to cause extensive environmental pollution. The International Alliance for Anti-Cancer (IARC) officially designated cadmium as an IA carcinogen in 1993 [1]. In addition, cadmium toxicity has a cumulative effect. The biological half-life of cadmium can last for 10–30 years when cadmium enters the human body, which causes long-term chronic toxicity [2]. The target organs of cadmium toxicity in humans include the heart, lungs, liver, kidneys, brain, and testis [3]. Cadmium is excreted through the kidneys, and therefore, they are the main target of toxicity. The cadmium toxicity to kidneys is particularly obvious and can cause apoptosis, necrosis, and atrophy of renal tubular epithelial cells [4].

However, the mechanism of how cadmium causes toxicity to kidneys is still unclear. Previous studies have shown that various complex cellular and molecular mechanisms are involved [5]. Among them, the classical theory holds that cadmium can produce oxidative stress and lipid peroxidation by interfering with redox reaction which can induce cell apoptosis [6]. Moreover, cadmium interferes with the steady state of basic metal ions, such as calcium ion signaling [7, 8], and interacts with metalloproteins to induce metallothionein expression [9]. In addition, cadmium interacts with nonmetallic proteins such as those containing active cysteine.

Phospholipase D1 (PLD1), a member of the PLD family, hydrolyzes membrane lipid phosphatidylcholine to produce choline and the second messenger lipid product phosphatidic acid (PA) [10]. It has been reported that PLD1 is related to cell survival signals and can inhibit the apoptotic process. Increased expression of PLD1 can prevent cell cycle arrest and apoptosis [11]. PLD1 activity is involved in multiple procedures in tumors, including proliferation, metabolism, angiogenesis, and metastasis. Increased PLD1 activity and expression have been reported in various tumor tissues and cell lines, and inhibition of PLD1 activity can slow down the growth and metabolism of tumors. This indicates that the oncogenic role of PLD1 activity is in promoting cell proliferation and inhibiting apoptotic processes [12–15]. PLD1 activity has been demonstrated in signal transduction, cell proliferation, and antiapoptotic processes [16]. In addition, studies in inflammation and autoimmune diseases have shown that PLD1 is associated with cell proliferation and cell apoptosis inhibition [17].

Recent studies demonstrate that PLD1 may be associated with toxicological mechanisms of various heavy metal ions. It was reported that mercury toxicity can activate PLD in vascular epithelial cells [18]. In addition, PLD1 expression decreased significantly in some kidney injuries [19]. Therefore, it is not difficult to see that PLD1 may be potentially related to the chronic toxicity of cadmium.

In this study, we explored the potential role of PLD1 in the pathogenesis of kidney injury induced by chronic cadmium exposure using proteomics and bioinformatics analysis. The low expression of PLD1 was implicated in cadmium-induced apoptosis of renal tubular cells.

## 2. Materials and Methods

### 2.1. Animal Experiments

Animal models were produced using a well-established protocol by chronic exposure to low-level Cd [20]. The animal experiment was conducted in compliance with the United States NIH Guide for the Care and Use of Laboratory Animals (National Research Council of the National Academies, 2011), and all studies were approved by the Institutional Animal Care and Use Committee of Southern Medical University.

Adult male Sprague-Dawley rats (weight of 220–240 g, 8–10 weeks old, *n* = 12) were purchased from Southern Medical University Laboratory Animal Center. All rats were kept in Specific Pathogen Free animal facilities under conditions of 21 ± 1°C and 50–80% relative humidity at all times and were maintained on a 12/12 h light/dark cycle. All the animals had free access to water and food. CdCl_2_ (Sigma-Aldrich, St. Louis, MO, USA), at a daily dose of 0.6 mg/kg/d for 5 days per week, was subcutaneously injected for 6 weeks or 12 weeks (*n* = 3 per group), while the control rats (*n* = 6) received daily injections of isotonic saline. The day before the deadline of every group experiment, rats were transferred from a normal feeding cage to a metabolic cage, and 24-hour urine samples were collected. At the end of the protocol, the animals were intraperitoneally anesthetized with sodium pentobarbital (30 mg/kg) and euthanized. Blood was collected from the abdominal aorta using a blood collection tube containing EDTA. Kidney tissues were harvested and stored immediately in cryogenic vials in liquid nitrogen [21].

### 2.2. iTRAQ Quantification and Data Analysis

SDT lysate homogenate solution (4% SDS, 100 mM Tris-HCl, 1 mM DTT, pH 7.6) was added to kidney tissue samples removed from liquid nitrogen. After ultrasonic lysing, they were centrifuged at 14,000 × *g* and 25°C for 40 min and the supernatant was collected. Protein was quantified using the BCA method. The 8-plex iTRAQ labeling, strong cation exchange (SCX) separation, and HPLC-MS/MS analysis were performed by Applied Protein Technology Co. Ltd. (Shanghai, China). Proteins were identified by Proteome Discoverer 1.4 (Thermo Fisher, MA, USA) using the search engine Mascot 2.2 (Matrix Science, London, UK) against the UniProt mouse database (http://www.uniport.org/, 76,417 entries, downloaded December 12, 2014) and the decoy database. A *t*-test was used to determine the difference of expression with the criterion set as *p* value < 0.05, fold change > 1.2, and occurrence in at least two of the three biological replicates. The entire iTRAQ proteomics raw data have been deposited in the public proteomics database iProX (Project ID: IPX0001166000, http://www.iprox.org/).

### 2.3. Determination of Cd Concentration in Biological Samples

The Cd concentration assay used inductively coupled plasma mass spectrometry (ICP-MS). Kidney tissue and urine were collected, weighed, and digested. Samples were digested in 1% HNO_3_. A standard solution was made with a cadmium standard for ICP-MS (Sigma-Aldrich). The methods were as described previously [22].

### 2.4. Detection of Molecular Biomarkers in Blood and Urine

Blood and urine samples were collected. Kidney injury molecule 1 (KIM-1), *β*2-microglobulin (*β*2-MG), N-acetyl-*β*-D-glucosaminidase (NAG), and creatinine levels were measured using ELISA kits (Cusabio Biotech, Wuhan, China). The values of biomarkers in urine were normalized to urinary creatinine. (The creatinine level and creatinine clearance are shown in Supplementary [Supplementary-material supplementary-material-1]). Intracellular PA was detected using ELISA kits (Yutong Biotech, Yancheng, China). All of the ELISA tests were done according to the manufacturer's instructions.

### 2.5. Cell Culture and Treatment

A human proximal tubular cell line derived from a normal kidney (HK-2) and rat renal tubular epithelial cell lines (NRK-52E) were purchased from KeyGen BioTech, Nanjing, China. DMEM/F12 (Gibco, CA, USA) was used for culturing HK-2, and DMEM (Gibco) was used for NRK-52E. Cell lines were treated with Cd for 48 h approaching IC50, which was 40 *μ*M for HK-2 and 8 *μ*M for NRK-52E. PA (40 *μ*M, Sigma-Aldrich) was introduced and kept for 48 h to observe its effect on apoptosis. Adenovirus vector was used for transduction and overexpression of PLD1. The concentration of fetal bovine serum was 10%. Cells were grown at 37°C in a humidified atmosphere with 5% CO_2_.

### 2.6. Western Blotting

Total protein was extracted from the cells by lysis with RIPA lysis buffer (Beyotime, Shanghai, China). The protein content of the lysate was then determined using a BCA kit (Beyotime, Shanghai, China) according to the manufacturer's protocol. Then, equal amounts of the protein lysate were separated by SDS-PAGE, and the separated proteins were transferred to a PVDF membrane (Millipore, MA, USA). The membranes were blocked with 5% BSA for 1 h and then incubated with primary antibodies overnight at 4°C. Primary antibodies were as follows: PLD1 (1 : 1,000, CST, MA, USA), *β*-actin (1 : 10,000, Proteintech, IL, USA), and cleaved Caspase-3 (1 : 500, CST, MA, USA). The immune complexes were then immunoblotted with an HRP- (horseradish peroxidase-) conjugated anti-mouse or anti-rabbit immunoglobulin G antibody (1 : 2000, Bio-Rad, CA, USA). Immunodetection was performed using enhanced chemiluminescence reagents (Bio-Rad). All experiments were repeated three times and quantitatively analyzed using ImageJ.

### 2.7. RNA and miRNA Extraction and qRT-PCR Analysis

Total mRNA was extracted from kidney tissues or cells using an RNAiso Plus Kit (Takara Biotech, Kyoto, Japan) and converted into cDNA. qRT-PCR was performed using the QuantStudio 6 Flex Real-Time PCR System (Thermo Fisher, MA, USA). The relative change of gene expression was determined using 2^-*ΔΔ*^Ct with *β*-actin as the internal reference. The specific primers for *PLD1* and *β*-actin gene (Synbio Technologies, Suzhou, China) are shown in Supplementary [Supplementary-material supplementary-material-1].

miRNA was extracted using an mirVana™ miRNA isolation kit (Ambion, Austin, TX) and converted into 1st-Strand cDNA (Tiangen, Beijing, China). The relative change of miRNA expression was determined using 2^-*ΔΔ*^Ct with U6 snRNA as the internal reference. The specific primers for miR-122-5p and U6 snRNA (Synbio Technologies, Suzhou, China) are shown in Supplementary [Supplementary-material supplementary-material-1].

### 2.8. Apoptosis Assays

Flow cytometry was performed for apoptosis using the Guava easyCyte HT system (Millipore). Apoptotic cells were assayed using an Annexin V-FITC/PI apoptosis detection kit (Dojindo, Kumamoto, Japan). The assays were performed according to the manufacturer's instructions.

### 2.9. Cell Viability Rate Assays

Cells were seeded in a 96-well plate with different treatments, and then 10 *μ*l of CCK-8 reagents (Dojindo) was added and incubated for 2 h. Then, the absorbance at 450 nm was measured using a microplate reader. All steps were conducted following the product manual.

### 2.10. Dual-Luciferase Reporter Gene Assays

A PLD1 3′UTR fragment was inserted into a psiCHECK-2 vector (Promega, USA) to build a psiCHECK-2-PLD1 3′UTR-wt construct. The psiCHECK-2-PLD1 3′UTR vector was nicked with Xho I enzyme (New England Biolabs, USA) and Not I enzyme (New England Biolabs). Q5 Site-Directed Mutagenesis Kit (New England Biolabs) was used to mutate in site 1761 psiCHECK-2-PLD1 3′UTR-wt to produce psiCHECK-2-PLD1 3′UTR-mut. NRK-52E and HK-2 cells were plated at a density of 1 × 10^5^ cells per well in a 96-well plate and allowed to attach for 24 h. The constructed dual-luciferase plasmid psiCHECK-2-PLD1 3′UTR-wt, psiCHECK-2-PLD1 3′UTR-mut, or the control luciferase plasmid (100 ng/well) was cotransfected with miR-122-5p mimics or NC mimics for 36 h using Lipofectamine 3000 reagent (Life Technologies, USA). Luciferase activity was assayed 36 h after transfection using a Dual-Luciferase Reporter Assay Kit (Promega, USA). Experiments were repeated five times.

### 2.11. Data Analysis

All experiments were repeated at least three times, and values are presented as mean ± standard deviation (SD). SPSS (version 22.0) was used for statistical analysis. Statistical significance was determined by using Student's *t*-test or two-way ANOVA. A value of *p* < 0.05 was considered statistically significant. GraphPad Prism 6 was used to draw the figures.

## 3. Results

### 3.1. Measurements of Kidney Impairment in Rats with Chronic Cd Exposure

**Figure 1:**
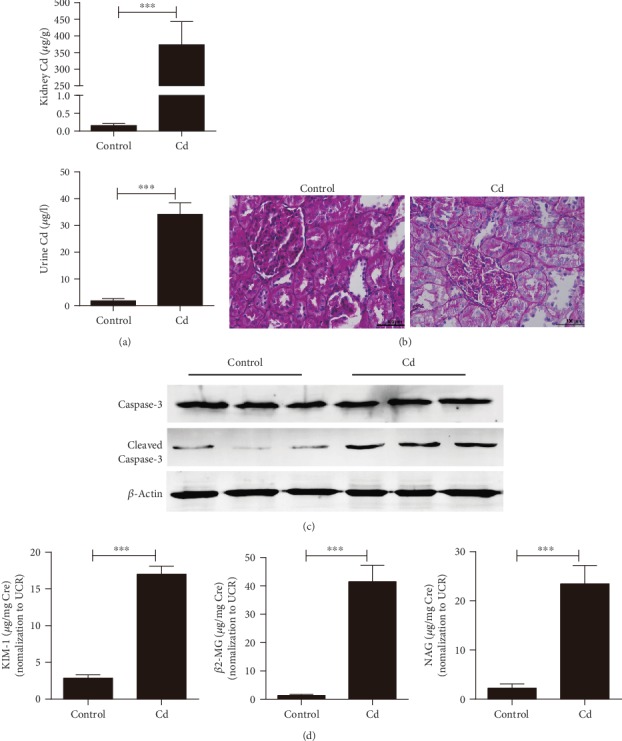
Measurements of kidney impairment in rats exposed to chronic Cd for 12 weeks. (a) The concentration of Cd in kidney tissue and rat urine detected by using ICP-MS. (b) Pathohistological changes in kidney tissue produced by Cd exposure (Periodic Acid-Schiff stain, HE ×400). (c) Cleaved Caspase-3 was increased after Cd exposure. (d) Early biomarkers of kidney injury in urine detected with ELISA. Rats exposed to CdCl_2_ at 0.6 mg/kg/d for 5 days per week for 12 weeks. Data were represented as mean ± SD, *N* = 3. ^∗^*p* < 0.05, ^∗∗^*p* < 0.01, and ^∗∗∗^*p* < 0.001.

Cd concentrations in rats were evaluated by ICP-MS (Figure 1(a)). In groups exposed for 6 weeks, cleaved Caspase-3, KIM-1, NAG, and *β*2-MG were not significant when compared with the control group although the Cd level was elevated in both kidneys and urine (Supplementary Fig. [Supplementary-material supplementary-material-1]). In addition to the rising Cd level, remarkable fibrosis of the nephric tubule was observed in the Periodic acid–Schiff (PAS) stained tissue of rats exposed to Cd for 12 weeks (Figure 1(b)). Simultaneously, high levels of renal cleaved Caspase-3 as well as urinary KIM-1, NAG, and *β*2-MG were observed in rats exposed to Cd for 12 weeks when compared to the control group (Figures 1(c) and 1(d)). These results suggested that 12 weeks of chronic exposure of Cd may be more appropriate for the evaluation of Cd-induced kidney impairments.

### 3.2. PLD1 May Correlate Strongly with Cd-Induced Chronic Kidney Impairment

**Figure 2:**
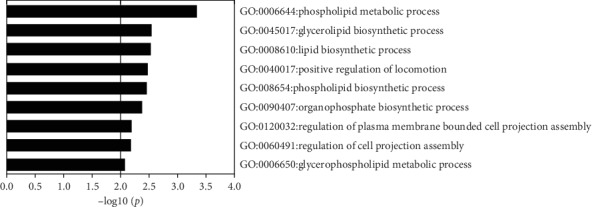
Pathway enrichment analysis of significantly altered proteins screened by proteomics of rat renal tissue after 12 weeks of cadmium exposure.

To fully understand the molecular signaling of Cd-induced chronic kidney impairment, the renal tissues of rats exposed for 12 weeks were analyzed by iTRAQ. Our analysis demonstrated that 49 proteins exhibited statistical alteration in the kidneys of rats exposed to Cd when compared to normal rats (fold change > 1.2 or <0.83, *p* < 0.05, Supplementary [Supplementary-material supplementary-material-1]). The data were then further assessed through pathway enrichment analysis using Metascape (http://www.metascape.org/). We found that quite a few of the terms were linked to PLD1, using *p* < 0.01 as the threshold. The top significant pathways are displayed in Figure 2. Among these pathways, four were involved in lipid metabolic processes, phospholipid metabolism (R-RNO-1483257, *p* = 0.0059), lipid biosynthetic process (GO: 0008610, *p* = 1.8 × 10^−7^), phospholipid metabolic process (GO: 0006644, *p* = 4.6 × 10^−4^), and phospholipid biosynthetic process (GO: 0008654, *p* = 3.4 × 10^−3^). Moreover, both Western blotting and qRT-PCR analysis of renal PLD1 expression exhibited a significant decline in Cd-exposed rats. These results strongly suggest that the renal damage caused by chronic Cd exposure might be associated with PLD1 expression.

### 3.3. PLD1 Expression Is Inhibited Significantly by Cd Exposure in Renal Tubular Cell Lines

**Figure 3:**
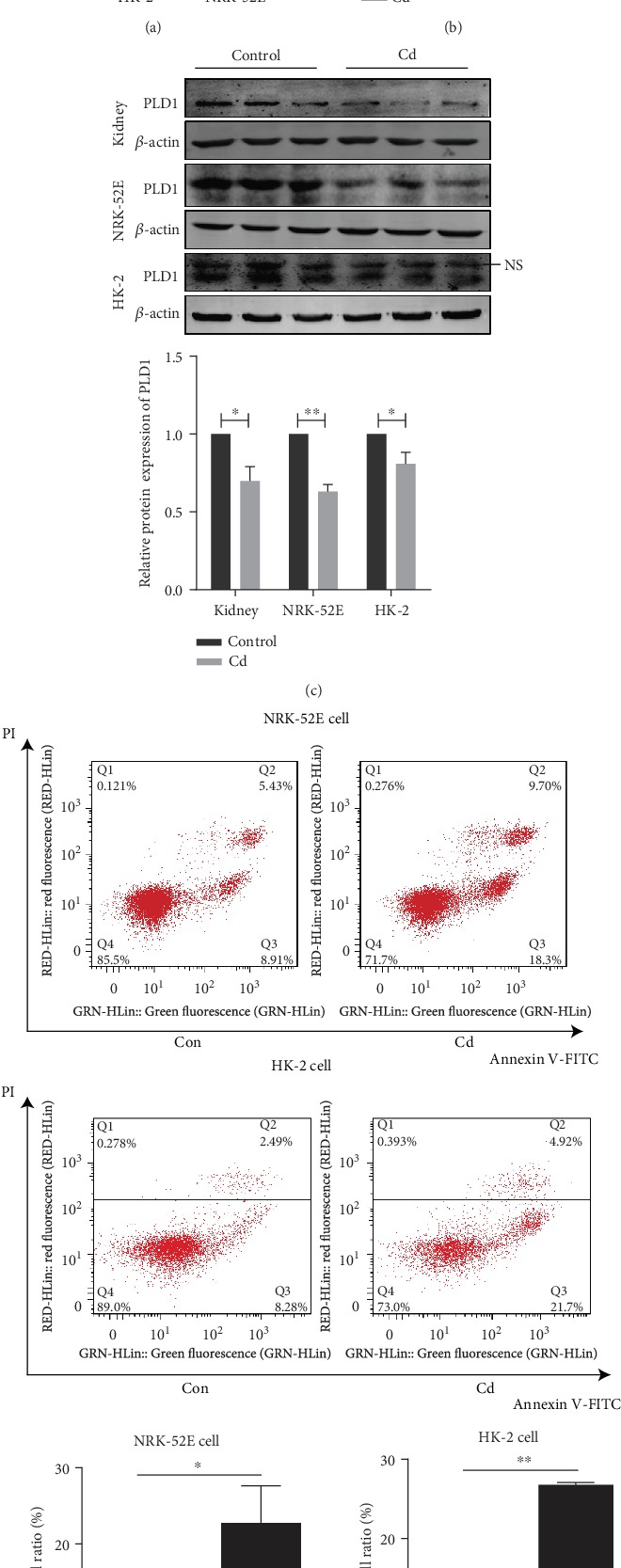
PLD1 expression is inhibited significantly by Cd exposure in renal tubular cell lines. (a) The viability of NRK-52E and HK-2 cells after Cd exposure measured by CCK-8 assay. (b) The mRNA expression of PLD1 was decreased after Cd exposure. (c) The protein expression of PLD1 was decreased after Cd exposure. (d) Apoptosis ameliorated in renal tubular cells exposed to Cd performed by flow cytometry. Con: control group. Rats exposed to Cd at 0.6 mg/kg/d for 5 days per week for 12 weeks. NRK-52E cells exposed at 8 *μ*M CdCl_2_ for 48 h, HK-2 cells exposed at 40 *μ*M CdCl_2_ for 48 h. NS: nonspecific band. Data are mean ± SD. ^∗^*p* < 0.05 and ^∗∗^*p* < 0.01.

Human-derived (HK-2) and rat-derived (NRK-52E) renal tubular cell lines were cultured with CdCl_2_ for a 48 h determination of the cell viability using a CCK-8 assay. As shown in Figure 3, the CCK-8 assay demonstrated that the cell viability of HK-2 and NRK-52E has declined approaching IC50 after CdCl_2_ exposure (Figure 3(a)). In both cell lines, the apoptosis cell numbers were elevated after CdCl_2_ exposure whereas the levels of PLD1 were reduced significantly in both protein and mRNA when compared with control groups (Figures 3(b)–3(d)). These results suggested that Cd-induced renal tubular impairment may be correlated with PLD1.

### 3.4. PLD1 and Its Downstream Product PA Protect Cd-Induced Renal Impairment

**Figure 4:**
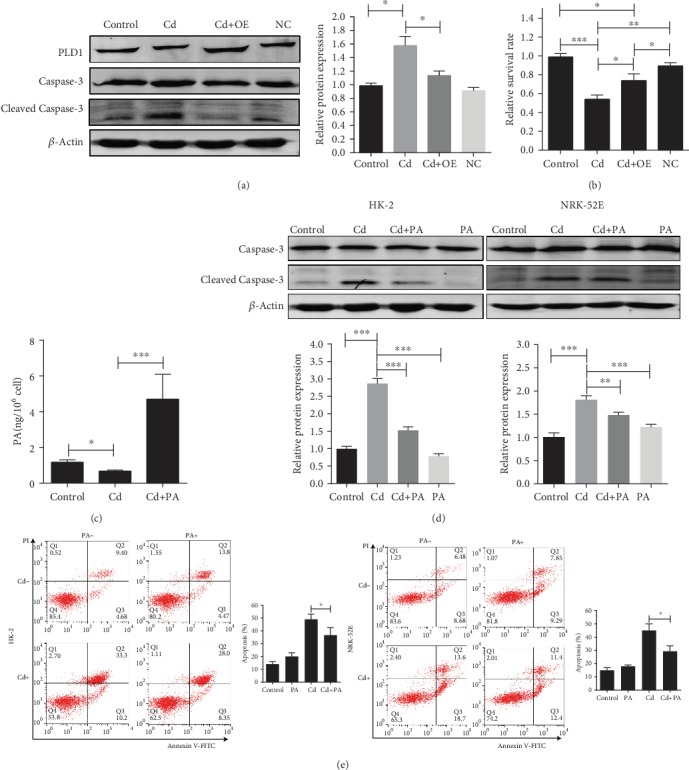
PLD1 and its downstream product PA protect cells from Cd-induced renal impairment. (a) Apoptosis was ameliorated after overexpression of PLD1 in HK-2 cells. (b) Cell survival rate was increased after overexpression of PLD1 in HK-2 cells assayed with CCK-8. (c) The concentration of PA in HK-2 cells detected with ELISA. (d) Apoptosis was ameliorated by PA treatment in HK-2 and NRK-52E cells. (e) Apoptosis was ameliorated by PA treatment detected in HK-2 and NRK-52E cells by flow cytometry. OE: overexpression of PLD1 with adenovirus vector. Data are mean ± SD from three experiments. ^∗^*p* < 0.05, ^∗∗^*p* < 0.01, and ^∗∗∗^*p* < 0.001.

To further understand the potential role of PLD1 in Cd-induced renal impairment, the PLD1-loaded ad-vector was transfected into HK-2 cells and then underwent Cd exposure. As shown in Figure 4, the level of cleaved Caspase-3 in cells overexpressing PLD1 was significantly declined compared to that in wild-type cells after Cd exposure (Figure 4(a)). Meanwhile, the CCK-8 assay displayed remarkably higher cell viability in cells overexpressing PLD1 after Cd exposure compared with wild-type HK-2 cells (Figure 4(b)). These data indicated that PLD1 may be a protector in Cd-induced renal tubular impairment.

In addition, the role of PA, a direct downstream product of PLD1, was revealed in HK-2 and NRK-52E cell lines after Cd exposure. Within the PA conducted, the level of cleaved Caspase-3 and cell apoptosis reduced significantly in the HK-2 and NRK-52E cell lines after Cd exposure (Figures 4(c)–4(e)). Taken together, these data suggested a positive role of both PLD1 and PA in Cd-induced chronic renal impairment.

### 3.5. The miR-122-5p Downregulates the Expression of PLD1 to Promote Apoptosis

**Figure 5:**
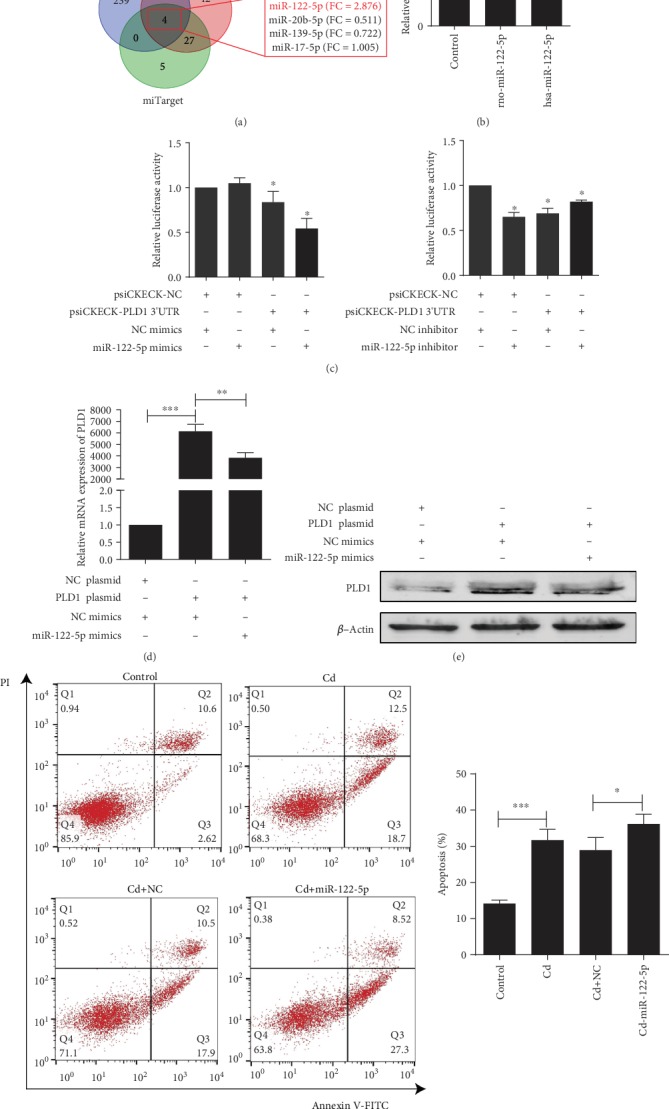
The miR-122-5p downregulates the expression of PLD1 to promote apoptosis. (a) Screening of candidate regulatory miRNAs for PLD1 in online databases (miRWalk, miRanda, and miTarget). (b) Expression of miR-122-5p was increased after Cd exposure in NRK-52E and HK-2 cells. (c) Regulatory effect of miR-122-5p on *PLD1* 3′UTR determined with dual-luciferase reporter assay. (d) miR-122-5p repressed mRNA expression of PLD1 while cotransfected with miR-122-5p and overexpression of PLD1 in NRK-52E cells. (e) miR-122-5p repressed protein expression of PLD1 in NRK-52E cells. (f) miR-122-5p promoted apoptosis in NRK-52E cells. Data are represented as mean ± SD, *N* = 3. ^∗^*p* < 0.05, ^∗∗^*p* < 0.01, and ^∗∗∗^*p* < 0.001.

As the PLD1 protein level was downregulated in both kidney tissues and proximal tubular cell lines exposed to Cd, we further examined the possibility that upregulation of miRNA might affect PLD1 expression during Cd exposure. Four candidate miRNAs that target the 3′-untranslated region (3′UTR) of PLD1 were searched in online databases (miRWalk, miRanda, and miTarget) and filtered based on scores and *p* values (Figure 5(a)). Among the four miRNAs, only miR-122-5p exhibited upregulation in the kidney tissues of rats exposed to Cd in our preceding miRNA profiling study; therefore, miR-122-5p was selected as the candidate regulatory miRNA. A qRT-PCR assay indicated that the expression of miR-122-5p was upregulated in both HK-2 and NRK-52E cells exposed to Cd for 48 h (Figure 5(b)). To determine whether a direct interaction occurs between miR-122-5p and PLD1 3′UTR, the wild-type PLD1 3′UTR reporter construct was cotransfected with a miR-122-5p mimic or inhibitor in NRK-52E cells in a dual-luciferase reporter assay (Figure 5(c)). The results indicated that miR-122-5p mimic could effectively reduce the luciferase activity while miR-122-5p inhibitor failed to do that. These results suggested that miR-122-5p regulated the expression of PLD1. Based on bioinformatics analysis, we presumed and validated by mutating the binding site that miR-122-5p regulates the expression of PLD1 by targeting position 1764–1784 in the 3′UTR of PLD1 mRNA (Supplementary Fig. [Supplementary-material supplementary-material-1]). The mRNA and protein expression of PLD1 was decreased significantly in cells cotransfected with a PLD1 overexpression construct and miR-122-5p (Figures 5(d) and 5(e)). NRK-52E cells transfected with miR-122-5p mimic were exposed to Cd; the apoptosis rate was significantly increased in the Cd with the miR-122-5p group compared with the Cd with the negative control using flow cytometry (Figure 5(f)). These results indicated that miR-122-5p downregulated the expression of PLD1, thereby promoting the occurrence of apoptosis.

## 4. Discussion

In the past few decades, the toxicology of cadmium has become an important public and environmental health issue [23]. Cadmium is one of the heavy metal ions that can be hoarded in mammals and result in the chronic impairment of organs, especially the kidneys [4, 24]. Nonetheless, the biochemistry and molecular mechanism of chronic renal impairment by Cd are still unclear. Therefore, in this present study, the chronic Cd exposure of rats and iTRAQ analysis were used to measure potential molecular factors of Cd-induced chronic renal impairment. The results suggested that PLD1 may be a potential protector in Cd-induced chronic renal impairment and the effectiveness of PLD1 and PA on chronic Cd exposure was rarely seen in recent investigations.

Numerous reports have demonstrated the renal impairment of chronic low-dose Cd exposure, and the detectable renal impairments after the same dose of Cd exposure became present at 9 weeks in rats [20]. Consistent with these reports, in our results, 6 weeks of Cd exposure enhanced the kidney and urinary Cd concentration, but no significant impairment occurred. As other investigators have reported that 12 weeks was the plateau for Cd-induced chronic renal impairment [20], the kidneys of rats exposed to 12 weeks of Cd were used to reveal the iTRAQ analysis.

In our iTRAQ analysis, ribosome biogenesis protein-1 (Bop-1) exhibited the most significant alteration (downregulation) in Cd-exposed rat kidneys. Nevertheless, Bop-1 was reported as a pivotal and terminal component for protein synthesis in mammals and was not disrupted in any of the fields. In contrast, PLD1 correlated with renal tubular dysfunction and chronic metabolic metal intoxication. Together with the downregulation of PLD1 in the kidneys of Cd-exposed rats, we, therefore, assumed that PLD1 has a key role in Cd-induced chronic renal impairments. As expected, PLD1 expression was inhibited in Cd-exposed renal tubular cell lines and the supplement of PLD1 attenuated the Cd-induced apoptosis of renal tubular cell lines.

PA is the metabolic product of PLD1 and is implicated in many cell processes [25]. It is established that PA is indispensable for autophagy by mediating autophagosome formation, which helps the cells to survive diverse stresses [26]. PA can also activate mTOR and help to suppress apoptosis by a mechanism involving complex downstream molecules [27]. Therefore, it is plausible that the downregulation of PLD1 and PA in proximal tubular cells induced by Cd may contribute to kidney injury. Moreover, PLD1 is easy to deactivate and degrade *in vivo* and is involved in the generation and stabilization of PA; therefore, PA may receive more attention in our future work.

It was reported that miR-122-5p expression was closely related with the apoptosis of breast cancer cells [28]. It was also found that miR-122-5p could inhibit the proliferation, invasion, and growth of bile duct carcinoma cells [29]. In this study, we found and verified that the expression of miR-122-5p was upregulated in HK-2 and NRK-52E cells after Cd exposure and binds to the 1761–1784 nt on the 3′UTR of PLD1. miR-122-5p can downregulate the expression of PLD1 to promote Cd-induced renal damage. Therefore, miR-122-5p may be a novel target for combating Cd-induced kidney injury.

However, the up- and downstream turnovers of Cd and PLD1 were not investigated in this study. Therefore, our future work will focus on the molecular mechanism of PLD1 on Cd exposure and more effective biofactors should be distinguished and measured.

Taken together, the results presented suggested that a strong correlation existed between Cd-induced chronic renal impairment and PLD1. Although the mechanism and sufficient relevant molecular interactions need to be validated, the protective role of PLD1 and PA on renal tubular cell lines indicated a novel perspective of chronic Cd exposure.

## Data Availability

The data used to support the findings of this study are included within the article.

## Abbreviations

PLD1: Phospholipase D1
PA: Phosphatidic acid
ICP-MS: Inductively coupled plasma mass spectrometry
iTRAQ: Isobaric tagging for relative and absolute protein quantification
KIM-1: Kidney injury molecule 1
*β*2-MG: *β*2-microglobulin
NAG: N-acetyl-*β*-D-glucosaminidase
HK-2: Human kidney 2
NRK-52E: Normal rat kidney 52E
DMEM: Dulbecco's modified Eagle's medium.

## Supplementary Materials

Supplementary Materials Suppl. Table 1: primers used for qRT-PCR in this study. Suppl. Table 2: serum and urine creatinine level and creatinine clearance of SD rats chronically exposed to a low level of Cd. Suppl. Fig. 1: measurements of kidney impairment in rats exposed to chronic Cd for 6 weeks. (A) The concentration of Cd, detected using ICP-MS, in kidney tissue and urine of rats. (B) Pathohistological stain in kidney tissue produced by Cd exposure (Periodic Acid-Schiff stain, HE ×400). (C) Cleaved Caspase-3 expression after Cd exposure. (D) Early biomarkers of kidney injury in urine detected with ELISA. Rats exposed to CdCl_2_ at 0.6 mg/kg/d for 5 days per week for 6 weeks. Data are represented as mean ± SD, *N* = 3. ^∗^*p* < 0.05 and ^∗∗∗^*p* < 0.001. Suppl. Fig. 2: miR-122-5p binding site in PLD1. (A) Schematic diagram of the miR-122-5p putative binding site in PLD1. (B) Validation of miR-122-5p binding site in PLD1 using dual-luciferase reporter assay. Suppl. Fig. 3: phospholipase D signaling pathway constructed by Pathview based on KEGG.
