# Supplementary material for: Phospholipase D1 Ameliorates Apoptosis in Chronic Renal Toxicity Caused by Low-Dose Cadmium Exposure
Source: Biomed Res Int. 2020 Mar 31;2020:7091053. doi: 10.1155/2020/7091053 (PMC7150706; doi:10.1155/2020/7091053)
Supplement: Supplementary Materials — Suppl. Table 1: primers used for qRT-PCR in this study. Suppl. Table 2: serum and urine creatinine level and creatinine clearance of SD rats chronically exposed to a low level of Cd. Suppl. Fig. 1: measurements of kidney impairment in rats exposed to chronic Cd for 6 weeks. (A) The concentration of Cd, detected using ICP-MS, in kidney tissue and urine of rats. (B) Pathohistological stain in kidney tissue produced by Cd exposure (Periodic Acid-Schiff stain, HE ×400). (C) Cleaved Caspase-3 expression after Cd exposure. (D) Early biomarkers of kidney injury in urine detected with ELISA. Rats exposed to CdCl2 at 0.6 mg/kg/d for 5 days per week for 6 weeks. Data are represented as mean ± SD, N = 3. ∗p < 0.05 and ∗∗∗p < 0.001. Suppl. Fig. 2: miR-122-5p binding site in PLD1. (A) Schematic diagram of the miR-122-5p putative binding site in PLD1. (B) Validation of miR-122-5p binding site in PLD1 using dual-luciferase reporter assay. Suppl. Fig. 3: phospholipase D signaling pathway constructed by Pathview based on KEGG. [file 7091053.f1.pdf]

Supplementary Material

Supplementary Tables

Suppl. Table 1 Primers used for qRT-PCR in this study

Gene names	Primers	Species
PLD1	F: ACAGCATTAGCAGCGTCG<br>R: CCAGTTGAACCCAGTCCTT	Rattus norvegicus
PLD1	F: CTTATCCTTTTCTGCTTCCA<br>R: AGTGCCTGCTGTTCTCTATC	Homo sapiens
β-actin	F: CCCATCTATGAGGGTTACGC<br>R: TTTAATGTCACGCACGATTTC	Rattus norvegicus
β-actin	F: GGGAAATCGTGCGTGACATTAAGG<br>R: CAGGAAGGAAGGCTGGAAGAGTG	Homo sapiens
miR-122-5p	F:AGTGGAGTGTGACAATGGT<br>R:CCAGTTTTTTTTTTTTTTTCAAACACC	Rattus norvegicus
miR-122-5p	F: AGTGGAGTGTGACAATGGT<br>R:CCAGTTTTTTTTTTTTTTTCAAACACC	Homo sapiens
U6	F: CTCGCTTCGGCAGCACA<br>R: AACGCTTCACGAATTTGCGT	Rattus norvegicus
U6	F: CTCGCTTCGGCAGCACA<br>R: AACGCTTCACGAATTTGCGT	Homo sapiens

F: forward primer, R: reverse primer

Suppl. Table 2 Serum and urine creatinine level, and creatinine clearance of SD rats chronically exposed to a low level of Cd

**Groups**	**SCr (mg/dL)**	**UCr (mg/dL)**	**CrCl (dL/min)**	p^*
Control	0.37±0.1	212.49±42.81	11.17±3.57	0.253
Cd treatment	0.32±0.05	232.58±47.21	13.82±3.90	

SCr, serum creatinine; UCr, urine creatinine; CrCl, creatinine clearance. N = 3, $^*t$-test for CrCl difference.

Supplementary Figures

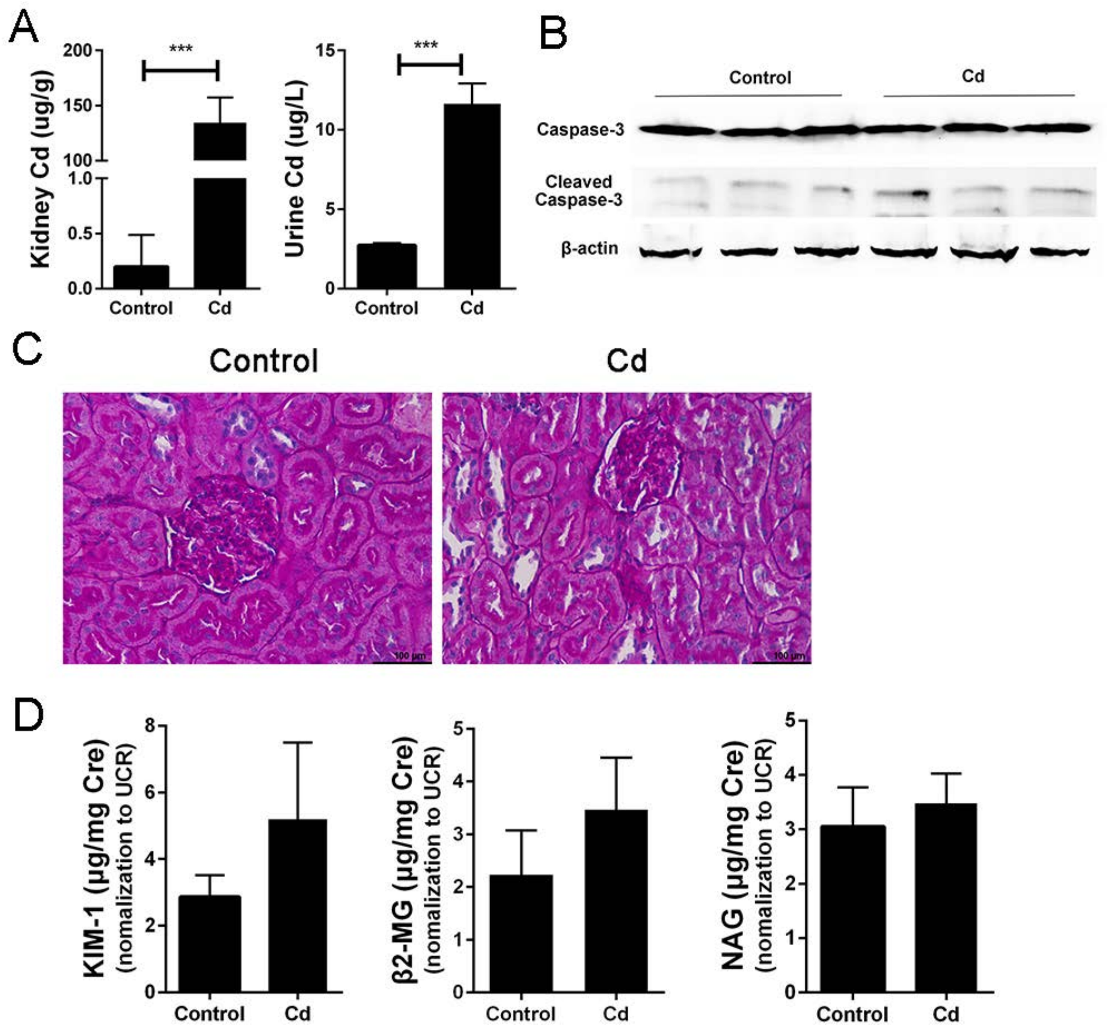

Suppl. Fig. 1 Measurements of kidney impairment in rats exposed to chronic Cd for 6 weeks. (A) The concentration of Cd, detected using ICP-MS, in kidney tissue and urine of rats. **(B)** Pathohistological stain in kidney tissue produced by Cd exposure (Periodic Acid-Schiff stain, HE×400). **(C)** Cleaved-Caspase-3 expression after Cd exposure. **(D)** Early biomarkers of kidney injury in urine detected with ELISA. Rats exposed to $CdCl_2$ at 0.6 mg/kg/d for 5 days per week for 6 weeks. Data are represented as mean ± SD, N = 3, $^{*}p < 0.05$, $^{***}p < 0.001$.

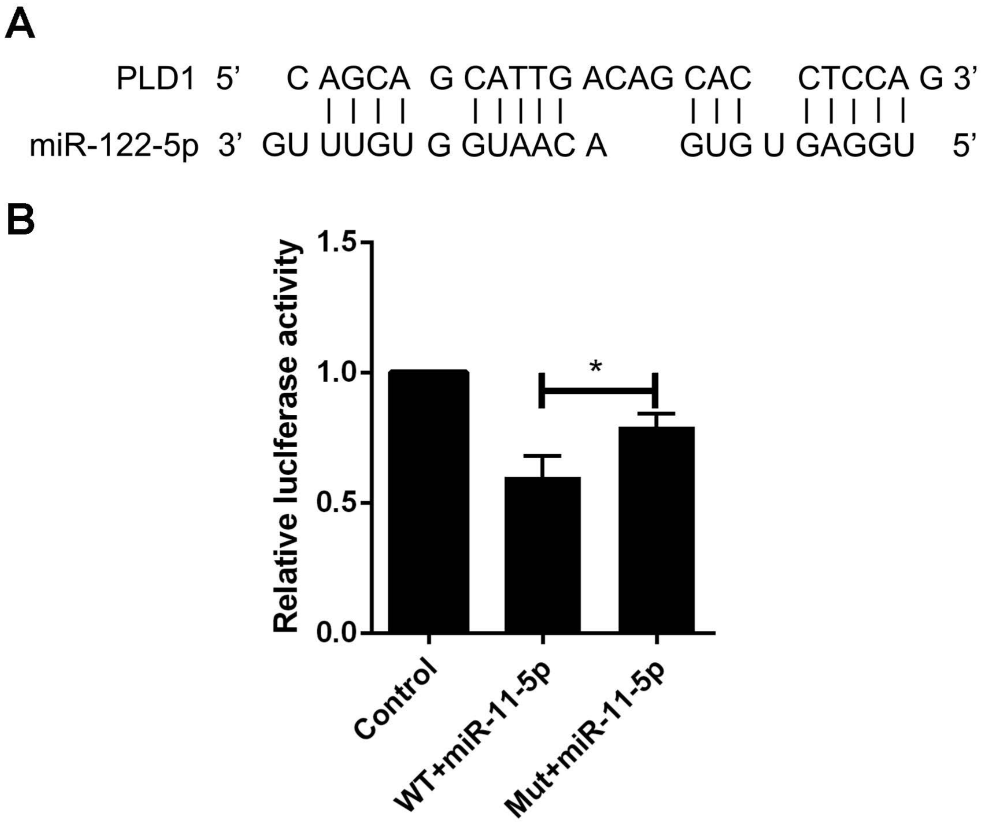

Suppl. Fig. 2. miR-122-5p binding site in *PLD1*. (A) Schematic diagram of the miR-122-5p putative binding site in *PLD1*. (B) Validation of miR-122-5p binding site in PLD1 using dual-luciferase reporter assay.

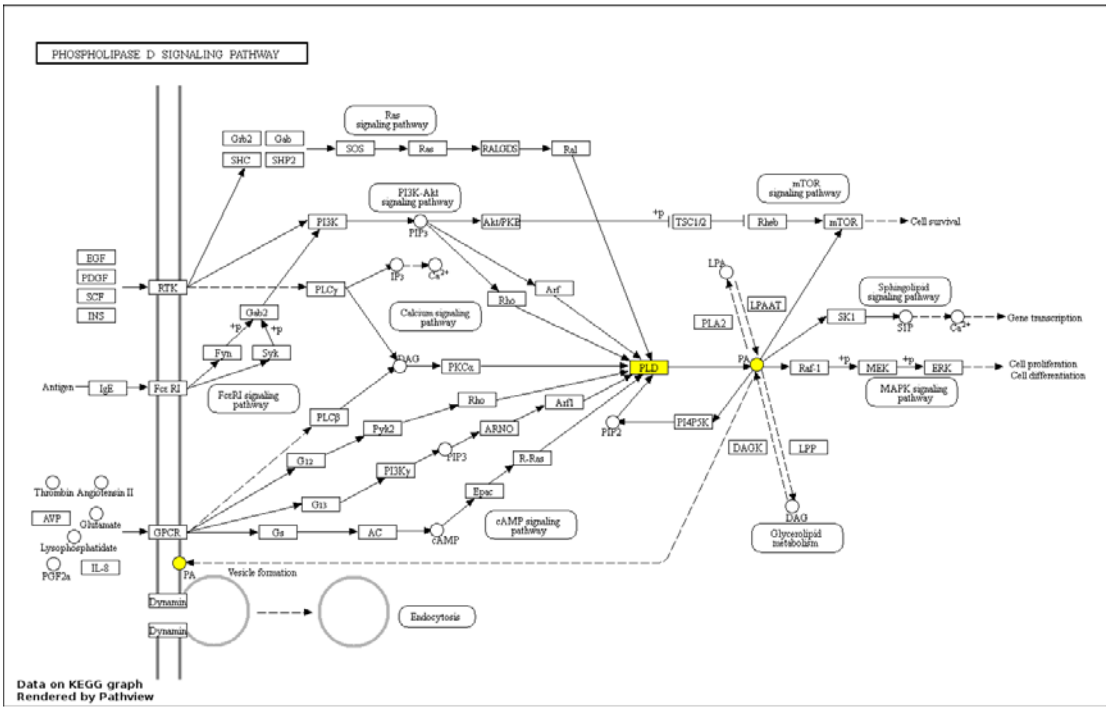

Suppl. Fig. 3 Phospholipase D signaling pathway constructed by Pathview based on KEGG.
